# Design of Anti-Eccentric Load Sensor for Engineering Operation Early Warning Based on Particle Swarm Optimization

**DOI:** 10.3390/s24165293

**Published:** 2024-08-15

**Authors:** Kaile Yu, Weizheng Ren, Yiran Zhang, Yutong Ge, Yuxiao Li

**Affiliations:** 1 School of Electronic Engineering, Beijing University of Posts and Telecommunications, Beijing 100876, China; ykl@bupt.edu.cn (K.Y.); zyr1999@bupt.edu.cn (Y.Z.); geyt@bupt.edu.cn (Y.G.); liyuxiao@bupt.edu.cn (Y.L.); 2 School of Modern Post, Beijing University of Posts and Telecommunications, Beijing 100876, China

**Keywords:** sensor, eccentric load, particle swarm optimization, early warning of engineering operation, eccentric load compensation

## Abstract

The accuracy of aerial work platform weighing is essential for safety. However, in practice, the same weight placed at different locations on the platform can yield varying readings, which is a phenomenon known as eccentric load. Measurement errors caused by eccentric loads can lead to missed detections and false alarms in the vehicle safety system, seriously affecting the safety of aerial work. To overcome the influence of eccentric load, the current engineering practice relies on multiple measurements at multiple points and averaging the results to eliminate the eccentric load, which greatly increases the work intensity of workers. To address the aforementioned issues, this paper proposes a three-dimensional force/torque shear force compensation scheme based on bending torque and torsional torque for pressure. The goal is to ensure that the sensor on the aerial work vehicle platform can accurately measure the anti-eccentric load under single-point measurement conditions. A three-box structure anti-eccentric load-weighing sensor for the aerial work platform was designed. Its structure has the advantages of high mechanical strength and no radial effect, ensuring the safety of aerial work, improvement of measurement sensitivity, and enabling of real-time and accurate acquisition of force/torque in three directions. In order to further improve the measurement accuracy of 3D force/torque compensation, a particle swarm optimization algorithm was adopted to optimize the 3D force/torque shear force compensation, thereby improving the safety of engineering operations. Through the verification of a self-made testing platform, the anti-eccentric load sensor designed in this study can ensure that the measurement error of objects at any position on the platform is less than 1.5%, effectively improving the safety of high-altitude platform engineering operations.

## 1. Introduction

With the increasingly complex working environment and work requirements of engineering operations in the fields of construction, fire protection, and emergency rescue and the growing demand for intelligent construction machinery, modern intelligent machinery has higher and higher requirements for force sensing and control. Accurate and rapid measurement is essential to ensure the stability and safety of the operation platform [1,2,3]. Therefore, the anti-eccentric load force sensor needs to realize accurate and fast sensing of the force and torque on the platform on the basis of taking into account the requirements of compact size and can provide reliable and fast feedback control. This kind of sensor should not only be able to work stably in harsh environmental conditions but also needs to adapt to various dynamic changes that may occur in high-altitude operation.

During the operation of the platform, the real-time weight feedback is the key to the safety and reliability of the early warning system. The conventional weighing system usually uses the single point weighing method. When the weighed object on the platform has a certain mass and the center of gravity of the weighed object is not in the center of the weighing platform, it will bring bias error to the early warning system, affect the weight feedback results of the early warning system, and may affect the safety of the whole production process. Therefore, it is urgent to develop a high-precision torque sensor to reduce the measurement error caused by eccentric load [4].

In previous works, various approaches have been proposed to enhance the accuracy and stability of electronic scales, particularly those utilizing multiple sensors. Guo, for instance, introduced a system scheme aiming to refine the precision and reliability of multi-sensor electronic scales by designing a dynamic compensation circuit based on the sensor’s input–output characteristic curve. This method significantly improved the weighing accuracy of the electronic scale [5]. Similarly, Jing employed curve fitting techniques to establish a sensor inverse model for nonlinear correction, integrating software and hardware solutions to enhance measurement accuracy [6]. However, these methods faced limitations due to nonlinear errors.

Advancements in load cell technology, such as the Profi Line IP44 OEM model product invented by Sartorius Corporation (Göettingen, Germany), offers high resolution and a wide measuring range, although it is more suited to precision weighing than large-scale applications. Li’s approach stood out by introducing an intelligent weighing error compensation method specifically for truck scales, utilizing a neural-network-based fusion method to improve accuracy, particularly for large-range weights [7]. Nevertheless, its applicability was primarily tested in a ground environment, which differs significantly from the more complex dynamic interference present in other work environments.

The above studies mostly conducted in-depth and innovative research on a certain aspect of the eccentric load sensor. However, in the actual development process of anti-eccentric sensor products, improving the accuracy of the sensor is a systematic problem, which requires a systematic solution to the sensor’s elastic structure design, weak signal acquisition and processing, and mass measurement offset compensation so as to effectively improve the measurement accuracy of the sensor [8,9,10]. Therefore, this paper proposes a new design scheme for eccentric load measurement sensors, adopting a three-box structural design. Measuring the torque in the bending and torsion directions to compensate for the measured pressure can eliminate the eccentric load. The aim is to ensure that the sensor on the aerial work vehicle platform can accurately measure the anti-eccentric load under single-point measurement conditions.

## 2. Problem Description

There is a growing demand for aerial work platforms. According to statistics, the annual demand for these platforms is approximately 16,000 in the United States, 21,000 in the European Union, around 6000 in Japan, and roughly 3000 in China. The high-altitude work vehicle is a specialized type of equipment designed for elevated tasks in fields such as construction engineering, power engineering, and emergency rescue operations. Typically, these vehicles feature a boom length that can reach up to 40 m [11]. When subjected to a load capacity of 1 t, the heavy object exerts a torque of 260,000 N·m. This torque, with its inherent rotational tendency, can potentially induce significant tilting forces during high-altitude maneuvers. To maintain engineering safety standards, an onboard safety system is equipped to trigger real-time alarms in case of overload on the high-altitude work platform. This precautionary measure serves to prevent the vehicle from becoming unstable or tilting due to excessive force. Therefore, the accuracy of weighing on high-altitude work vehicle platforms is of utmost importance to ensure the safety of high-altitude operations.

**Figure 1:**
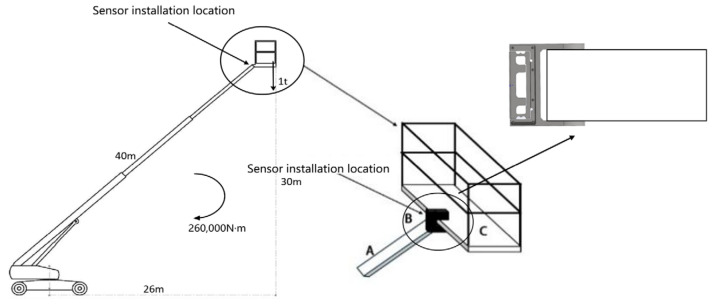
Force model diagram of a high-altitude operation vehicle.

Figure 1 shows the force model of a common high-altitude work vehicle: A is the boom of the aerial work vehicle, B is the anti-eccentric load sensor, and C is the platform of the aerial work vehicle. From the connection method in Figure 1, it can be seen that weighing platform C perceives and measures the weight of the load through the shear force experienced by the anti-eccentric load sensor B and feeds real-time shear force information back to the high-altitude operation warning system.

**Figure 2:**
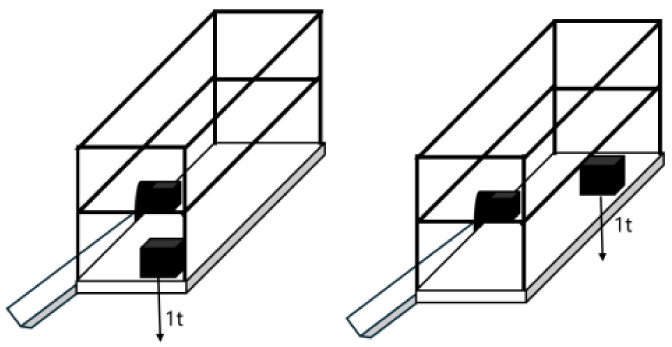
Renderings of weight placement positions.

This procedure of placing a 1 t load weight on the left side of the platform, subsequently relocating the same weight to the right side of the platform, and maintaining an equivalent distance from the edge allows for the measurement and comparison of weighing results at different positions, enabling the determination of measurement error. The weight placement method during the weighing test is shown in Figure 2. After measurement, it was found that placing a 1 t standard weight in different positions can produce a weighing error of 50 kg. Due to the width of high-altitude work platforms, when an object is placed at the edge of the platform, it will generate shear forces in two directions—torsional shear, and transverse bending—which will decompose the pressure on the platform and affect the weighing results. In engineering, this interference force is collectively referred to as the eccentric load force, and the existence of the eccentric load force is the main reason for the weighing deviation of the platform. In practical engineering operations, measurement errors caused by eccentric loads can cause missed or false alarms in the onboard safety system, seriously affecting the safety of high-altitude operations.

Currently, there is a scarcity of compensation algorithms specifically designed for eccentric load in weighing sensors on aerial platforms. The mainstream method still relies on the least squares method for compensation. However, due to the complexity of the aerial work platform system and its varying work environments, such linear algorithms struggle to achieve ideal measurement accuracy. When exploring nonlinear compensation algorithms, particle swarm optimization (PSO), ant colony optimization (ACO), and whale optimization algorithm (WOA) have all been widely applied. Compared to the other two algorithms, PSO stands out due to its simple principle, efficient convergence speed, and low computer memory requirements, making it more suitable for practical engineering needs. The ant colony optimization has complex parameter configurations, and improper parameter selection may miss high-quality solutions. The emerging whale optimization algorithm, although innovative, is also affected by parameter selection and requires experimentation and adjustment to achieve optimal performance.

Therefore, this study aimed to solve the problem of platform weighing bias by using a three-dimensional force/torque shear force compensation scheme based on the bending direction torque and torque direction torque to pressure. The particle swarm optimization algorithm was used to further optimize the compensation parameters and improve measurement accuracy. An anti-eccentric force sensor for engineering operation warning was designed, which can accurately and stably measure the load weight at any position on the platform, enabling the engineering operation warning system to accurately alarm and improve the safety of engineering operations.

The engineering operation early warning system is a commonly used detection device in the operation process of aerial work vehicles at present, which can feed back the operation information such as load, tilt angle, and environmental conditions in real time so as to ensure that staff can work safely at heights [12,13]. However, in the process of the engineering operation, the eccentric load force generated by the uneven force point of the platform will affect the accuracy of the real-time load value fed back by the early warning system, resulting in the failure of real-time alarm for platform overload, resulting in the safety problems of platform subversion, cargo damage, and even personal injury [11]. Therefore, overcoming the eccentric load force of the platform is an urgent problem to be solved in the current engineering operation early warning system.

## 3. System Architecture Design

The anti-eccentric load sensor is mainly composed of a mechanical–electrical conversion module, signal acquisition module, and data calculation module.

The mechanical–electrical conversion module mainly consists of a sensor box elastomer and strain measurement bridge. The strain gauge constituting the strain bridge is pasted in the strain area of the elastomer. When the elastomer is subjected to external force, the strain area will deform, driving the strain gauge to deform, so as to change the resistance of each bridge arm of the measurement bridge and generate differential measurement signals for a Wheatstone bridge.

The signal acquisition module includes precision acquisition, control kernel, and data transmission. The precision acquisition unit collects the weak signal from the Wheatstone bridge, accurately amplifies the collected weak signal to 0.5–4.5 V voltage signal, and then converts it into a digital signal. The control core unit acquires the digital signal sent by the precision acquisition unit and processes and packs the data to the data transmission unit according to the predetermined protocol. The data transmission unit is responsible for sending the data frames provided by the control kernel unit to the upper computer data calculation module through different transmission modes.

The data calculation module is responsible for the calibration, calculation, analysis, and other related processing of the data sent by the lower computer and for processing the eccentric load error through data compensation.

## 4. Design of the Mechanical–Electrical Conversion Module

### 4.1. Design Analysis of the Sensor Elastomer

In the design of a sensor, the design of the sensing terminal or elastomer is very important. The quality of the elastomer directly affects the measurement accuracy of the sensor. The main factors affecting the quality of the elastomer are the size, sensitivity, and structure of the elastomer. In this study, the finite element analysis method was used to calculate the stress and strain distribution of elastomer in the process of loading and unloading so as to verify the scientificity of the structural design and to ensure that the size and the sticking position of the strain gauge met the requirements.

#### 4.1.1. Elastomer Structure

**Figure 3:**
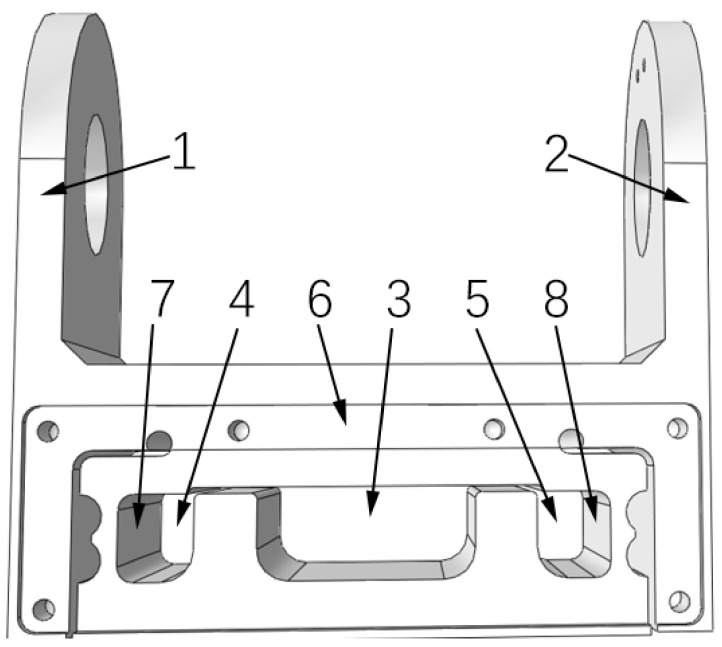
Elastic beam structure of the biased load force sensor used in the biased load platform. 1~2, fixed connector; 3, central main chamber; 4, left chamber; 5, right chamber; 6, box body; 7~8, strain-sensing area.

In order to make the sensor meet the characteristics of high-precision measurement and strong stability, the weight-weighing sensor of the aerial work platform designed in this study adopts the box structure so that the sensor is fixed with the aerial work platform through the upper two “ear-type” hole frames. The interior of the elastomer box type is divided into three chambers, with the acquisition system and the adapter terminal being placed in the middle main chamber and the strain gauge being pasted on the left and right chambers. On both sides of the left and right chambers are box-type elastomer deformation sensing parts, on which strain gauges form a strain bridge to sense the pressure *F*, bending force direction, and torsion direction moments *M*_*X*_ and *M*_*Y*_ in three-dimensional space. The structure has the advantages of high mechanical strength, no radial effect, and easy calibration. Its structure is shown in Figure 3, and the sensitive force/moment coordinate system is the right-hand coordinate system.

#### 4.1.2. Finite Element Analysis of Elastic Body

After the structure of the elastomer was determined, ABAQUS software was used for finite element analysis of the elastomer. Considering the contradiction between the range and sensitivity, it is especially affected by the thickness and length of the sensing piece. The sensitivity is usually positively correlated with the length of the sensing slice and negatively correlated with the thickness, while the range is just the opposite. Therefore, by continuously adjusting the volume, sensitivity, overload capacity, and other factors, the optimal elastomer size parameters were obtained. These parameters were used as inputs for ABAQUS finite element analysis to verify the stress and strain distribution during loading and unloading so as to ensure that the elastomer structure design was reasonable and could meet the performance requirements of the sensor.

The elastomer of the sensor is 2A12-T4 hard aluminum alloy with high hardness and strong fatigue resistance. Its elastic modulus is 73.4 × 10^9^ Pa, and its Poisson’s ratio is 0.33.

Leading enterprises in the industry, such as SANY Heavy Industry and Zoomlion Heavy Industry, adopt high-machine connecting arms with a diameter of 40 mm and a platform connection thickness of 180 mm. It is necessary to ensure that the outer half-ring of the connecting hole has a sufficient width to achieve the required connection strength. Therefore, the half-ring width was set to 20 mm, and the connection thickness was 10 mm. Based on these parameters, the designed box-type body size is 200 mm × 80 mm × 50 mm, which meets the structural requirements of the industry.

In the design of the sensor cavity, the smaller the cavity is, the higher the stiffness of the sensor. Considering that the internal circuit board size is 80 mm × 70 mm and that the wiring space and height need to be reserved, we set the size of the main cavity to 90 mm × 80 mm × 20 mm. This design can not only meet the placement requirements of the circuit board but also ensure the stiffness of the sensor. The dimension parameters of the elastic beam box-type part are shown in Table 1.

**Figure 4:**
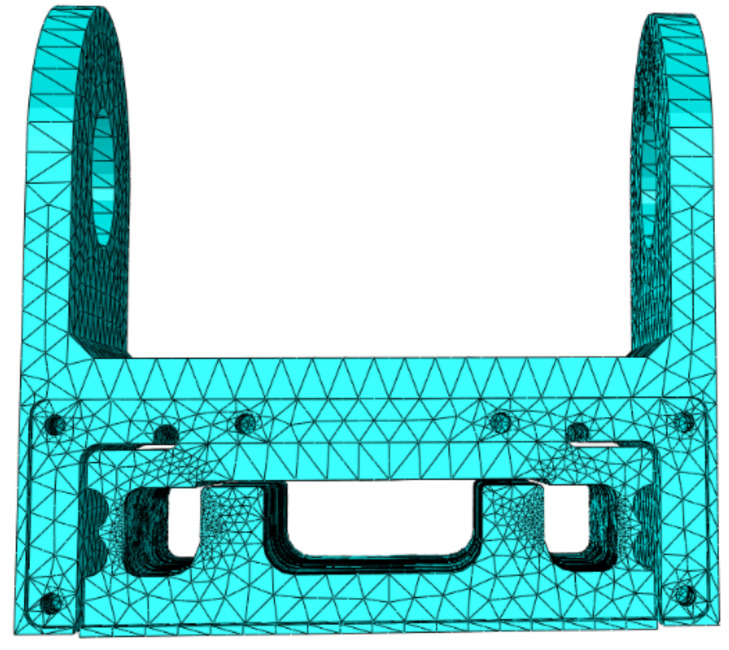
Elastomer finite element model.

SolidWorks 2020 was used to establish the elastomer model, abaqus2021 was imported for finite element selection and meshing, and the c3d10 high-precision solid element was selected. The generated finite element model is shown in Figure 4.

After the establishment of the finite element model of elastic body, it is necessary to carry out the load loading and solution calculation and analyze the strain after the stress. First, the constraint of the elastomer is set. Since the elastomer is fixed with the platform through the screw holes on the six wheel flanges behind the box shell, the XYZ degrees of freedom of the six screw holes are set to 0.

**Figure 5:**
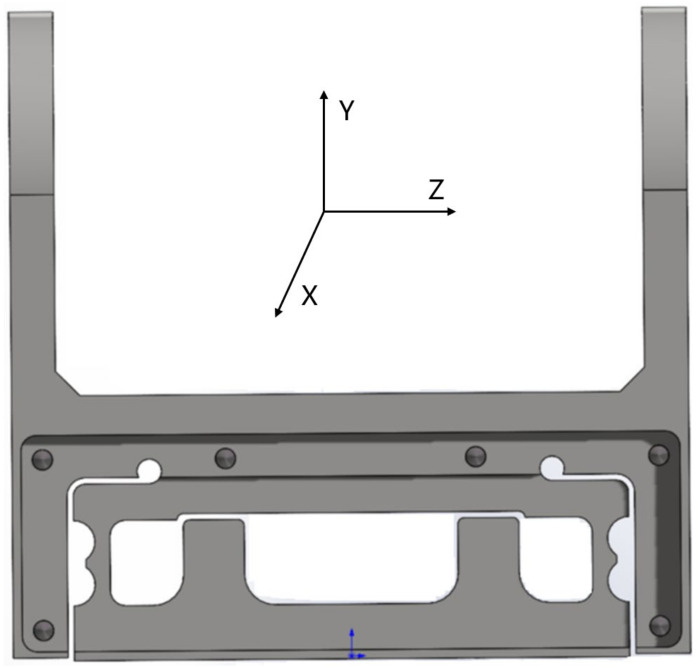
Diagram of sensor force direction.

Figure 5 is a diagram of the sensor force direction, where the positive direction of the *Z*-axis is the direction of the applied force *F*, the clockwise direction around the *X*-axis is the positive direction of *M*_*X*_, and the clockwise direction around the *Y*-axis is the positive direction of *M*_*Y*_.

**Figure 6:**
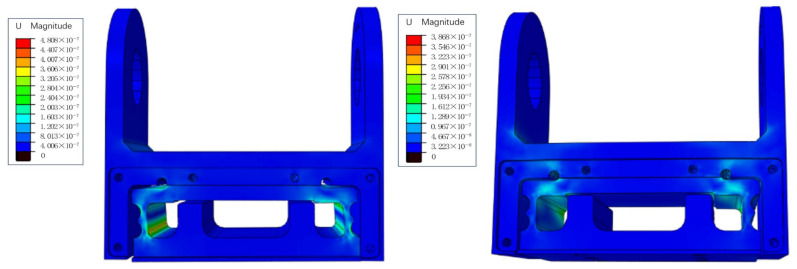
Strain diagram of the elastic beam after loading with *F* = 12,000 N or *M*_*Y*_ = 2400 N·m.

The loading analysis is carried out in three directions of *F*, *M*_*X*_, and *M*_*Y*_, and the loading value is determined by the design requirements. Under the design and application background of this paper, the measurement range of the sensor is *F* = ±12,000 N and *M*_*X*_ = *M*_*Y*_ = ±2400 N·m, and the overload capacity is *F* = ±20,000 N and *M*_*X*_ = *M*_*Y*_ = ±4000 N·m. The results for solving each dimension loading separately are shown in Figure 6.

**Figure 7:**
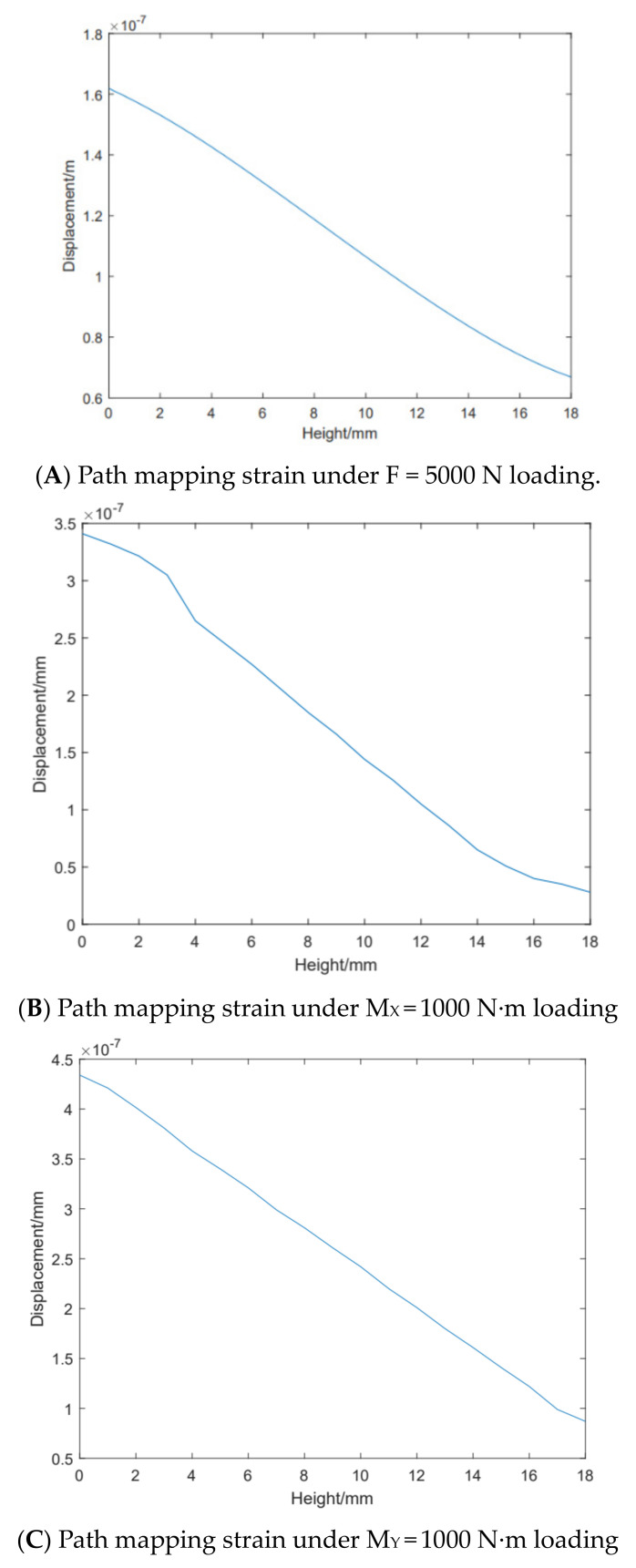
Path–mapped strain under loads of *F* = 5000 N, *M*_*X*_ = 1000 N·m, and *M*_*Y*_ = 1000 N·m.

In order to determine the accurate patch position of resistance strain gauge, it is necessary to analyze the corresponding relationship between the strain size of strain sensing area and the patch position. Using the path mapping technology of ABAQUS2018 software, the path is set to the height of the strain sensing area, and the strain of the strain sensing area is mapped to the path when the force is applied. The solutions are obtained separately for three loads, which are *F* = 5000 N, *M*_*X*_ = 1000 N·m, and *M*_*Y*_ = 1000 N·m. Diagram A in Figure 7 is the path mapping strain diagram under *F* = 5000 N, Diagram B in Figure 7 is the path mapping strain diagram under *M*_*X*_ = 1000 N·m, and Diagram C in Figure 7 is the path mapping strain diagram under *M*_*Y*_ = 1000 N·m. In the figures, the horizontal axis represents the height of the strain sensing area, and the vertical axis represents the strain size of the strain sensing area.

Since *M*_*X*_ and *M*_*Y*_ are used as compensation parameters to eliminate eccentric load errors, priority is given to the path mapping strain under pressure F loading when attaching strain gauges. Based on the results shown in Figure 7, the position of the strain gauge can be determined. Within the range of 4 to 14 mm in height of the strain sensing area, the linear relationship between height and strain size is the best, resulting in more accurate output. Meanwhile, the path mapping under *M*_*X*_ and *M*_*Y*_ loading satisfies the linear condition within the range of 4 to 14 mm. In order to improve the sensitivity and accuracy of the sensor as much as possible, the position of the strain gauge should be 4~14 mm high in the strain-sensing area.

**Figure 8:**
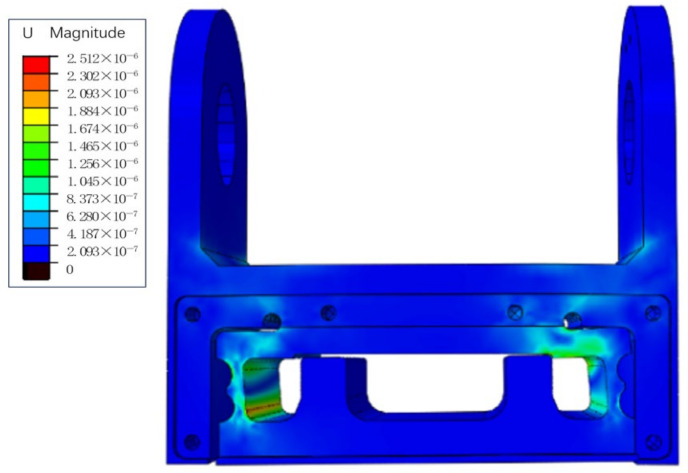
Elastic beam strain diagram under an extreme condition.

In order to ensure that the stress of the elastic beam under the limit of multi-dimensional force is within the allowable stress range of the material, it is necessary to simultaneously load full-scale forces or moments in three directions on the elastic beam. The strain of the elastic beam is shown in Figure 8.

The maximum strain on all paths is 2.124 × 10^−6^, ε × E = (2.124 × 10^−6^) × 73.4 GPa ≈ 162.507 KPa, which is less than the yield strength of 2A12-T14 duralumin alloy 325 MPa, indicating that the structure meets the strength design requirements.

### 4.2. Sensor Measurement Bridge Design

A Wheatstone bridge is one of the most common bridge networks/circuits, which can be used to measure resistance very accurately. A Wheatstone bridge is usually used with sensors to measure physical quantities, such as temperature, pressure, and strain [14]. The anti-eccentric load sensor designed in this study changes the resistance value of the strain gauge in the Wheatstone bridge under the stress state, which changes the input voltage, converts the external force signal into voltage signal, and obtains the force condition of the sensor.

**Figure 9:**
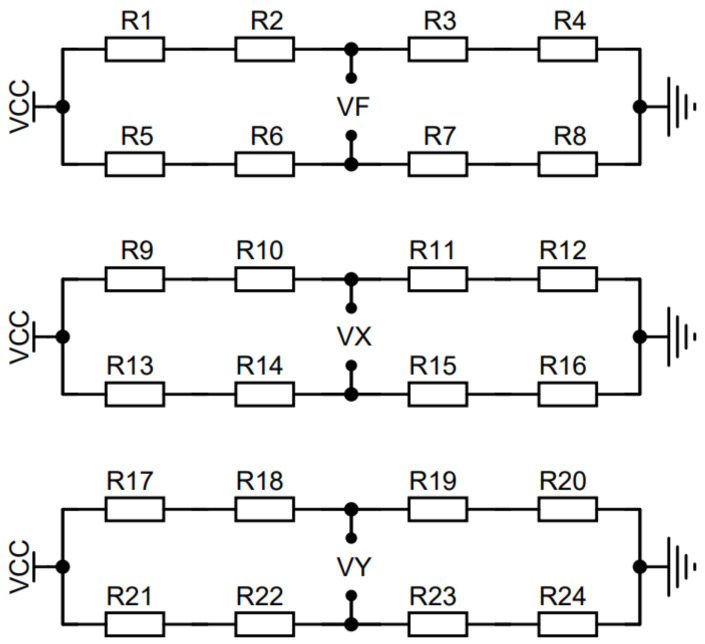
Circuit diagram of the three-way bridge group.

The measuring bridge of the anti-eccentric load box type force sensor adopts the full-resistance-strain Wheatstone bridge, and each force/torque direction corresponds to a fourth-order arm full bridge circuit. According to the analysis of the position of the corresponding strain gauge patch, the final strain gauge bridge circuit is shown in Figure 9. As shown in the figure, in order to obtain better sensitivity, the reference voltage of the V_R_ full bridge circuit provides a stable 24 V reference voltage for V_R_ through the boost circuit even when the power supply voltage of the si*x*-axis force sensor is 5 V. V_F_ is the pressure output signal, V_X_ is the bending torque output signal, and V_Z_ is the torque output signal.

## 5. Design of Signal Processing Unit

### 5.1. Precise Signal Acquisition Scheme Design

The Wheatstone bridge differential voltage signal is usually in millivolt level when the sensor is subjected to external force. In order to further improve the sensitivity of the sensor and consider the performance requirements of the subsequent A/D conversion circuit, we selected the A/D conversion chip Cs1237-SO (LCSC Electronics, Shenzhen, China) to amplify and collect each weak force/torque signal [15].

Cs1237-SO can efficiently convert tiny voltage signals into digital signals with 24-bit accuracy. The module signal input of the chip can accept differential signals, and a programmable operational amplifier is built in to effectively amplify the weak signals at the input. In addition, the chip is also integrated with a temperature sensor, which is of great significance to dealing with the nonlinearity and temperature drift between the magnitude and actual stress of the differential signal output by the strain bridge [16].

Cs1237-SO uses a simple and efficient two-wire SPI communication mode. Through SPI daisy chain communication, three digital signals can be transmitted to the control kernel module. This configuration effectively optimizes the signal acquisition and transmission and ensures the accurate capture and subsequent processing of weak signals. Therefore, the circuit system integrated with Cs1237-SO chip not only improves the sensitivity but also takes into account the accuracy and stability of the signal.

### 5.2. Control Scheme Design

In order to meet the requirements of high-speed dynamic performance and the multi-threaded communication of the anti-eccentric load box-type force sensor, the system uses stm32f103c8t6 as the system control processor, which has strong computing and control capabilities. The microcontroller uses armcortex-M3 core, the main frequency can reach 72 MHz, and the peak interpolation efficiency is 1.25 DMIPS/MHz. At the same time, it supports a variety of communication interfaces including SPI, I2C, USART, etc., which can receive all converted digital signals in real time and quickly and transmit the data compensated by the algorithm to the upper computer in real time.

### 5.3. Data Transmission Scheme Design

RS485, renowned for its exceptional interference resistance, dependable data transmission properties, and extended transmission range, has emerged as the preferred communication protocol for large outdoor machinery, such as high-altitude platforms. Its robust capabilities facilitate uninterrupted data flow in diverse and challenging environments, thereby offering substantial backing to the communication needs of outdoor machinery [17,18,19].

Therefore, the sensor RS-485 serial port communication mode in this study was used for the communication data transmission of system testing and platform weighing. The design purpose of the communication module is to transmit the data of the three force and torque components measured by the anti-eccentric force sensor to the external equipment for real-time monitoring, control, or further processing. By collecting the output signal of the sensor and through appropriate signal processing and data packaging, the data are encapsulated into RS-485 data frame for transmission.

## 6. Research on the Eccentric Load Compensation Algorithm

### 6.1. Three-Dimensional Force Unbalanced Load Compensation Scheme

This study achieved three-dimensional force compensation by acquiring calibrated point pressure, bending force, torque, and unbalanced load distance. A ratio calculation method is used to compensate for the nonlinear characteristics of the strain-type anti-unbalanced load force sensor, realizing the initial calibration of the unbalanced load force sensor. The calculation method is as follows.

**Figure 10:**
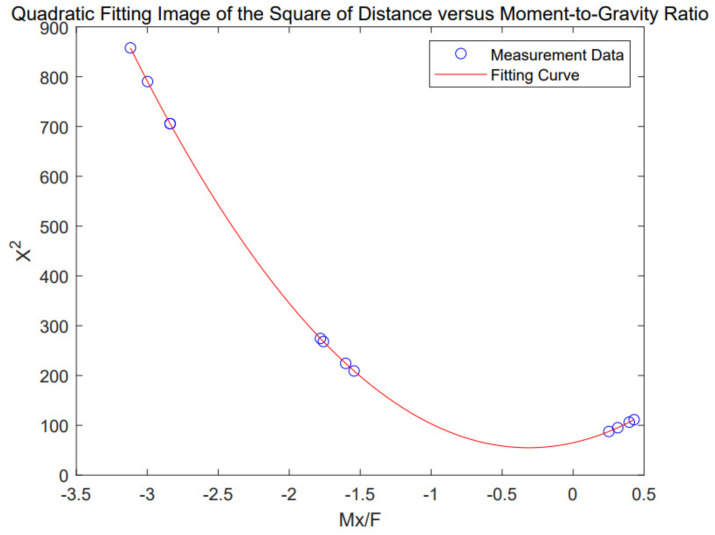
Quadratic fitting image of the square of distance versus moment−to−gravity ratio.

Since the eccentric force generated during the measurement process is caused by the gravity of the object and because it is found that the eccentric force at the same position is proportional to the weight of the object during the measurement, the position of the object on the plane can be determined by the ratio of the eccentric forces in two directions of the object to the gravity, namely *M*_*x*_/*F* and *M*_*y*_/*F*. Due to the slight deformation of the platform under the action of gravity, it can be seen from Figure 10 that the square of the distance and the mass moment in that direction can be fitted into a quadratic function relationship.

The ratio of the voltage data of the torque $M_{x}$ in the bending direction and the pressure direction f determines the eccentric load distance of the weight in the X direction, as shown in Formula (1).

(1) $$X^{2}=a_{2}{\left(\frac{M_{x}}{F}\right)}^{2}+a_{1}\left(\frac{M_{x}}{F}\right)+a_{0}$$

In the formula, X is the offset distance in the direction of bending force, and $a_{2},a_{1}{,and\;a}_{0}$ are the quadratic coefficients, first-order coefficients, and constant terms of the fitting function, respectively.

The bias load distance of the weight in the Y direction is determined by the voltage data ratio of the torque $M_{Y}$ in the torque direction and the pressure direction F, as shown in Formula (2).

(2) $$Y^{2}=b_{2}{\left(\frac{M_{y}}{F}\right)}^{2}+b_{1}\left(\frac{M_{y}}{F}\right)+b_{0}$$

In the formula, Y represents the offset distance in the direction of torque, and $b_{2},b_{1}{,and\;b}_{0}$ are the quadratic coefficients, first-order coefficients, and constant terms of the fitting function, respectively.

The weight compensation formula of the load cell is shown in Formula (3):

(3) $$M=Fk_{0}-\left(k_{1}X+k_{2}Y\right)F$$

where M is the gravity measurement value after compensation; $k_{0},k_{1}$, and $k_{2}$ are the compensation coefficients; and the calculation results of $k_{0},k_{1}$, and $k_{2}$ directly affect the weighing accuracy of the anti-eccentric load sensor, which is the core parameter to determining the performance and quality of the sensor.

### 6.2. Unbalanced Load Compensation Based on PSO Optimization

After the initial calibration, the compensation coefficient is further optimized and reconstructed by the PSO algorithm, and the sensor PSO is improved to evaluate the output weight m of the measurement system after eccentric load compensation through the fitness function. The PSO fitness function must be determined in advance. In this paper, the two norm between the weight value of the system output and the calibrated real weight value is selected as the fitness function of PSO. The calculation formula for the fitness function is Formula (4).

(4) $$f=\sum_{i=1}^{I}{\left(G_{i}-M_{i}\right)}^{2}$$

In the formula, f is the fitness function value, $M_{i}$ is the weight value of the system output for the i-th measurement, i is the number of measurements, and $G_{i}$ is the actual output of the i-th neuron.

In order to avoid the standard particle swarm optimization algorithm not taking into account the mutual influence between individual particles, this paper applies an improved particle swarm optimization algorithm, as shown in Formula (5):

(5) $$\left\{\begin{array}{c} x_{id}^{t+1}=\omega x_{id}^{t}+c_{1}r_{1}\left(P_{ad}-x_{id}^{t}\right)+c_{2}r_{2}\left(G_{best}-x_{id}^{t}\right)\\ P_{ad}=\frac{1}{m}\sum_{i=1}^{m}P_{bestid} \end{array}\right.$$

where $x_{id}^{t}$ is the position of the individual in the t-th iteration; ω is the inertia weight; $c_{1}$ and $c_{2}$ are the influencing factors of an individual’s “self-awareness” ability and “social cognition” ability, respectively; $r_{1}$ and $r_{2}$ take a random number between [0, 1]; $P_{bestid}$ is the individual optimal; $G_{best}$ is the global optimum; and $P_{ad}$ is the average optimal value for all individuals.

To enhance the global search performance of the algorithm and quickly escape from local optima, the inertia weight ω is set as a random number obeying a certain distribution. This is beneficial for meeting the requirements of refined search, avoiding flying over the optimal solution space, and also conducive to satisfying the requirements of particle global search, preventing the algorithm from falling into local optima and premature convergence and accelerating the convergence speed of the algorithm. The calculation formula of inertia weight ω is shown in Formula (6).

(6) $$\omega =\mu_{min}+\left(\mu_{max}-\mu_{min}\right)\cdot rand()+\sigma \cdot randn()$$

where $\mu_{min}\;and\mu_{max}$ are the minimum and maximum values of the average inertia weight, respectively; randn() is a uniform distribution function, which has equal probability of obtaining the optimal value, the maximum value, and the minimum value within the interval. The influence of the uniformly distributed weight ω is limited by $\mu_{max}-\mu_{min}$. the influence degree randn() is a normal distribution function; and σ is the variance.

The formula for the change in influencing factors is Formula (7) [20]:

(7) c1=c1ini−c1ini−c1finitTmaxc2=c2ini−c2ini−c2finitTmax

where $c_{1ini}$ and $c_{2ini}$ are the initial values of the learning factors $c_{1}$ and $c_{2}$, respectively; $c_{1fini}$ and $c_{2fini}$ are the final values of the learning factors $c_{1}$ and $c_{2}$, respectively; t is the current number of iterations; and $T_{max}$ is the maximum number of iterations.

### 6.3. PSO Algorithm Parameter Settings

**Figure 11:**
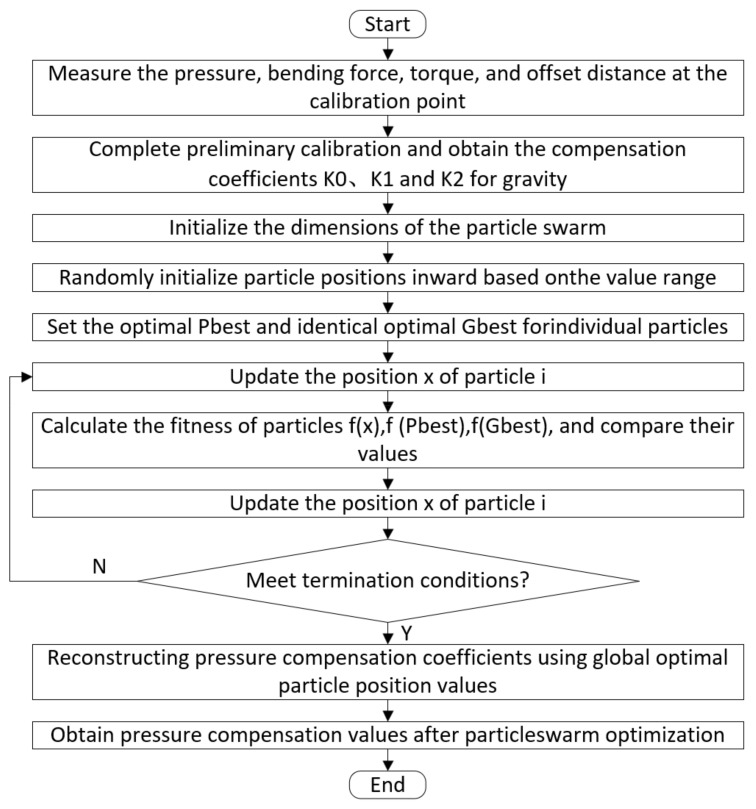
Flow chart of the experiment simulation based on PSO.

Based on the PSO algorithm for weighing compensation of bias force sensors, the basic parameters of the PSO algorithm needed to be set first. The experimental settings were as follows: 6 particles; 2000 iterations; upper and lower limits of position of −1000 and 1000, respectively; upper and lower limits of velocity of −3 and 3, respectively; a particle dimension of 9; learning factors of $c_{1}$ = 1.5 and $c_{2}$ = 1.5; a momentum learning rate of 0.03; and a total target error and convergence discrimination accuracy of 0.002. The specific process is shown in Figure 11.

## 7. Calibration Testing and Error Analysis

### 7.1. Static Calibration Testing

**Figure 12:**
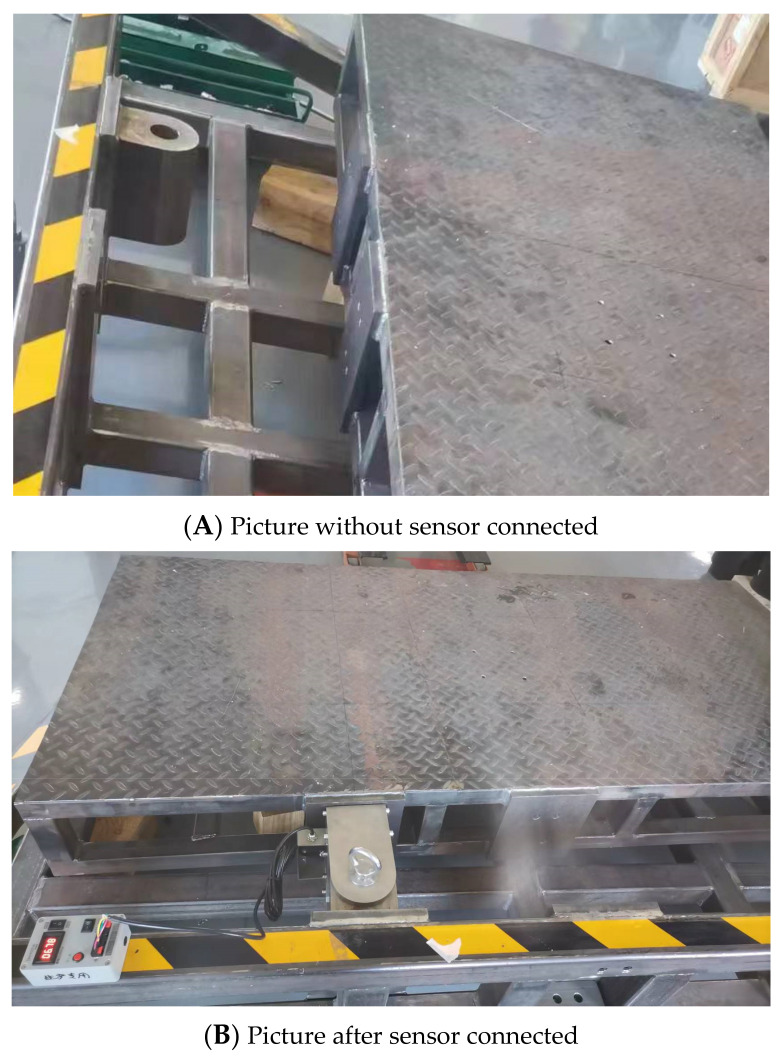
Two pictures before and after the connection of the calibration platform and sensor.

This study used a self-made bias load platform calibration device for static calibration. The platform can make the sensor sense the force/torque in three directions of pressure, bending force, and torque under the condition of loading heavy objects. The calibration process is simple, and the force value is stable. In the experiment, three directional force values were obtained by placing weights at different positions on the platform, with weights with M1 level accuracy being used (the relative error of the weight shall not exceed 0.001%). The connection between the calibration platform and the sensor is shown in Figure 12. In Figure 12, Diagram A shows the platform before sensor connection. It is connected and fixed via the simultaneous insertion of an iron axle through the holes in the box sensor ears and the platform frame, which is the same fixing method as that used in aerial work platforms. Diagram B shows the platform after being connected with the iron axle. At the same time, the material and thickness of the test platform are consistent with those of the aerial work platform of Sany Heavy Industry, ensuring versatility. The test environment was at a room temperature of 25 degrees Celsius, and the experimental environment was close to the actual work site.

Weight 1 generates a calibration force in the negative *X*-axis direction, weight 2 generates a calibration force in the positive *Z*-axis direction, and weight 3 generates a calibration torque in the negative *M*_*Y*_ direction. The method of generating forces/torques in other directions is similar. This calibration device can generate forces/moments in multiple directions simultaneously, facilitating the testing and experimentation of combined forces/moments.

Using a simulation high computer platform, the mass of standard weights of 100 kg, 200 kg, and 300 kg were placed on the sensor. Each weight was placed on the platform for six tests, and the six measurements were placed at different positions. The average of 20-voltage signal measurements was taken as calibration data for the same measurement. Table 2 shows the small millivolt voltage signals and offset distance generated by the placing of different mass weights at different positions on the platform. The data in the table were used as calibration data for the bias force sensor.

From Table 2, it can be concluded that the *X* offset torque *M*_*x*_ will approximately decrease linearly with the increase of the *X* direction offset distance, the *Y* offset torque *M*_*Y*_ will approximately decrease linearly with the increase of the *Y* direction offset distance, and the pressure *F* will approximately increase linearly with the increase of the weight. Therefore, the offset compensation scheme can be satisfied by the ratio of torque to pressure.

### 7.2. Three-Dimensional Force-Biased-Load Compensation

The collected data are transmitted to the upper computer display panel through an RS-485 communication module, and the first-order linear function relationship between pressure F and weight mass M is obtained through three-dimensional force-biased-load compensation. The voltage signal value can be converted into a mass measurement value through calculation. Due to the self-weight of the measurement platform, the data are reset before calculation and analysis, and the net weight of the measured weight is calculated after resetting. Table 3 shows the mass measured by the data measured in Table 3 under the conversion of the upper computer program.

From the table, it can be seen that the platform only calculates the load mass based on the first-order linear function relationship between pressure F and weight mass M, which will cause an error of about 6% due to the interference of eccentric load force. At the full range of 1200 kg, the eccentric load error gravity will generate an additional torque of about 20,000 N·m on the crane, which will seriously affect the safety of the crane operation. Therefore, a three-dimensional force system measurement is required to eliminate errors caused by biased loads through bending force and torque direction torque.

Based on the data from the 19 calibration points above, combined with Formulas (4)–(6), the parameter data were fitted using MATLAB 2021 software to obtain the sensor compensation coefficient. The data are recorded in Table 4.

Table 5 shows the comparison data before and after implementing three-dimensional bias load balancing, using the substituted 19-calibration-point data into Equations (4)–(6).

From the table, it can be seen that the pressure measured by the bending direction torque and torque compensation platform can effectively reduce the error caused by bias load. The measurement error can reach 3%, which is about 2% higher than that before equilibrium. This improves the safety of engineering operations to a certain extent. In order to further reduce measurement errors, this study used the particle swarm optimization algorithm to optimize the compensation coefficient.

### 7.3. Particle Swarm Optimization

The compensation coefficients were optimized through the particle swarm optimization process described in Section 6.3 of this article. Table 6 shows the compensation coefficients obtained from the calibration point data after particle swarm optimization.

From Table 7, it can be seen that after the compensation parameters were optimized through particle swarm optimization algorithm, the measurement error was approximately below 1.3%. Compared with before equilibrium, the measurement error increased by about 5%. Compared with after equilibrium, the measurement error increased by about 1.5%, effectively improving the measurement accuracy of the calibration point.

**Figure 13:**
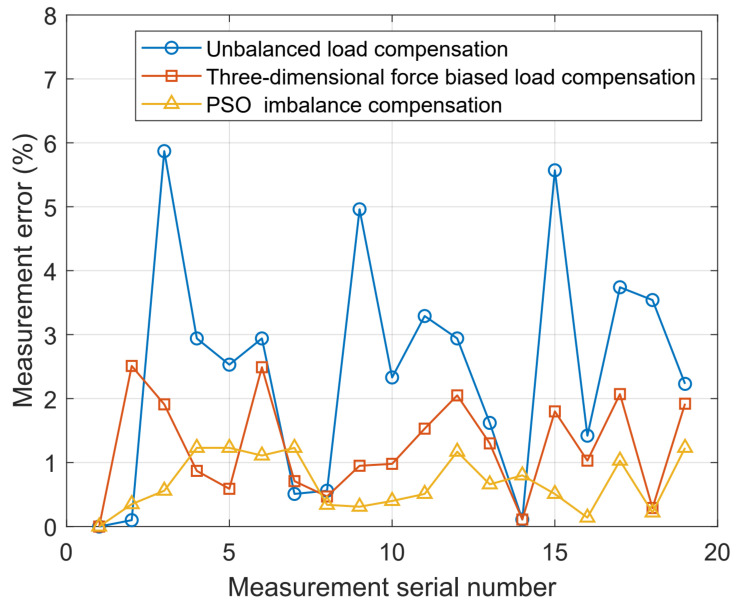
Weighing error graphs for the three different methods.

Based on the analysis of Figure 13, it is apparent that the PSO compensation algorithm significantly diminishes the measurement errors arising from imbalance forces at calibration points in comparison to the other two algorithms.

### 7.4. Precision and Error Analysis

In order to further test the accuracy of the anti-eccentric load sensor under the particle swarm optimization algorithm, six measurements were randomly selected at non-calibration points to demonstrate the universality of the measurement effect of the sensor and its algorithm designed in this study. The quality and location of random point measurements are shown in Table 8.

Weighing tests were conducted on the random point weights in Table 8 using three methods: no bias load compensation, three-dimensional force bias load compensation, and particle swarm optimization bias load compensation. The measurement results are shown in Table 9.

The measurement errors of the three weighing calculation methods presented in Table 9—namely, no bias load compensation, three-dimensional force bias load compensation, and particle swarm optimization bias load compensation—were calculated. The calculation results are shown in Table 10.

**Figure 14:**
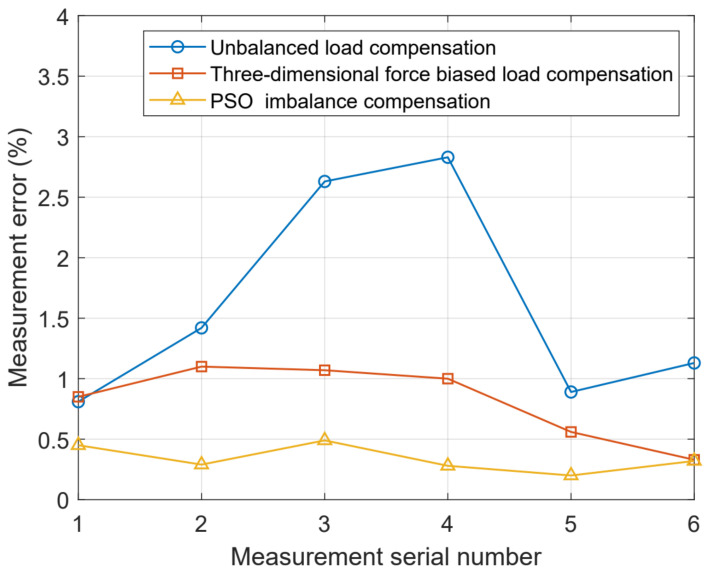
Weighing error graphs for three different methods.

Based on Figure 14, it is evident that the particle swarm optimization PSO imbalance compensation algorithm significantly mitigates the measurement errors induced by imbalance forces in comparison to the other two algorithms. Based on Table 10, the anti-eccentric load sensor based on particle swarm optimization bias compensation algorithm has an improvement of about 2% in random point position error compared to unbiased load compensation and about 0.6% in three-dimensional force bias compensation. In summary, the anti-eccentric force sensor based on the particle swarm optimization algorithm for engineering operation warning can effectively reduce measurement errors caused by bias force and improve the safety of engineering operations.

## 8. Case Application Experiments

**Figure 15:**
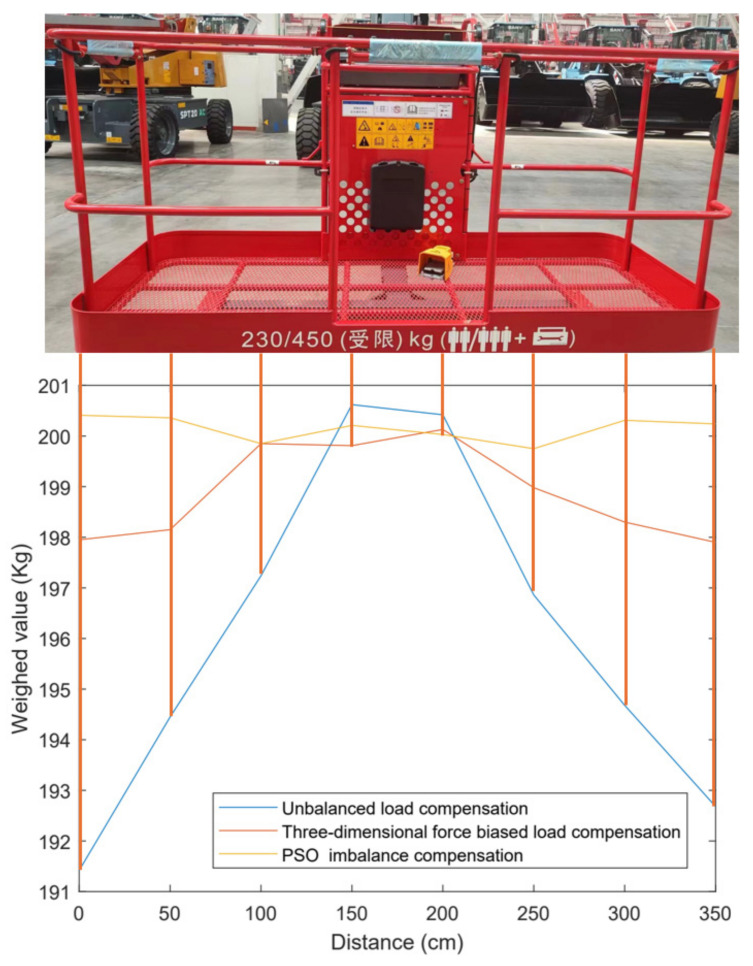
Load test diagram of a high platform.

To further test the usability of the anti-eccentric load sensor for engineering warning, in this study, the anti-eccentric load sensor was applied to the aerial work platform. To test the effect of the sensor designed in study paper in overcoming the eccentric load on the aerial work platform, the calibration process described above was completed on a 350 cm wide aerial work platform and placed a 200 kg counterweight at different positions of the aerial work platform for load testing. Figure 15 shows the load test diagram of placing weights at different positions on the aerial work platform.

As can be seen from the figure, compared to the state where the sensor was not subjected to bias load balancing, the anti-eccentric load sensor optimized based on particle swarm optimization had a relatively stable and accurate weighing curve at different positions, and there was no numerical decrease in the weighing mass at either of the platform due to bias load. This proves that the three-dimensional force/torque gravity compensation algorithm based on particle swarm optimization effectively overcomes the error caused by bias load force.

The actual test results show that the anti-eccentric load sensor can accurately measure the force and torque on the high-altitude work platform simultaneously, ensuring the reliability of the high-altitude work vehicle warning system. This application not only confirms the performance and availability of sensors but also provides important data support for the safety system of high-altitude work vehicles.

## 9. Conclusions

In response to the challenge posed by bias errors in force sensors due to bias force, which hinder the identification, prediction, and response to potential engineering risks, we propose several innovative designs. These designs incorporate anti-bias load structural elastomers; high-gain, high-linearity, and precise acquisition; and processing based on multi-channel weak differential signals. Additionally, we introduce a three-dimensional force/moment gravity compensation scheme based on the particle swarm optimization algorithm. To validate our designs, we conducted calibration and testing using a customized high-altitude work platform fixture. By comparing sensor test results after applying the particle swarm optimization with measurement accuracy before bias load balancing, we observed a significant enhancement in the sensor’s resistance to bias load and a substantial improvement in the platform’s weighing accuracy. These advancements ensure the reliability and safety of the engineering operation warning system. The experimental outcomes confirm the efficacy of our proposed methods. Furthermore, the successes achieved in this design establish a robust foundation for future in-depth research on six-dimensional force sensors.

Despite notable advancements in key technologies, such as the anti-bias load sensors developed in this study, there is still considerable scope for improvement in process optimization, compatibility with various data transmission modes, and testing and calibration systems. Through this design process, valuable experience has been gained in the development of measurement sensors for security systems, and areas requiring further refinement have been identified. Simultaneously, this experience has clarified the direction for future efforts, marking a significant learning milestone.

## Figures and Tables

**Table 1:** Dimension parameters of the elastic beam box-type part (unit: mm).

	Box Body	Central Main Chamber	Left and Right Chambers	Strain Sensing Area
Length	200	90	20	80
Width	80	80	80	30
Height	50	20	20	

**Table 2:** Data for the three-dimensional micro millivolt voltage signals under different load weights and orientations.

No.	Mass (kg)	Eccentric Load Distance in *X* Direction (mm)	Eccentric Load Distance in *Y* Direction (mm)	The Electrical Signal Generated by Pressure *F* (mV)	The Electrical Signal Generated by Torque *M*_*x*_ (mV)	The Electrical Signal Generated by Torque *M*_*Y*_ (mV)
1	0	0	0	4.41	5.61	−4.92
2	100	0	170	7.69	5.77	−7.59
3	100	−100	710	7.50	7.52	−12.93
4	100	530	180	7.79	1.21	−7.80
5	100	540	690	7.61	1.83	−13.31
6	100	1050	170	7.79	−3.98	−7.86
7	100	1010	665	7.71	−4.23	−13.11
8	200	−110	280	10.95	7.66	−12.12
9	200	−100	640	10.66	8.29	−19.83
10	200	640	200	11.14	−6.26	−11.52
11	200	650	650	10.77	−4.23	−20.47
12	200	1020	180	11.18	−14.73	−11.34
13	200	1010	665	10.88	−12.82	−21.03
14	300	−80	250	14.28	8.09	−15.62
15	300	−70	680	13.73	9.29	−28.75
16	300	640	250	14.42	−12.24	−15.92
17	300	630	660	13.91	−9.64	−28.25
18	300	1090	270	14.63	−26.31	−16.43
19	300	1020	650	14.06	−21.83	−29.07

**Table 3:** Test platform standard weight-weighing error.

No.	Mass (kg)	The Electrical Signal Generated by Pressure *F* (mV)	Eccentric Load Distance in *X* Direction (mm)	Eccentric Load Distance in *Y* Direction (mm)	Measured Data (kg)	Measurement Error
1	0	4.41	0	0	0.00	0%
2	100	7.69	0	170	99.90	0.10%
3	100	7.50	−100	710	94.13	5.87%
4	100	7.79	530	180	102.94	2.94%
5	100	7.61	540	690	97.47	2.53%
6	100	7.79	1050	170	102.94	2.94%
7	100	7.71	1010	665	100.51	0.51%
8	200	10.95	−110	280	198.89	0.56%
9	200	10.66	−100	640	190.08	4.96%
10	200	11.14	640	200	204.66	2.33%
11	200	10.77	650	650	193.42	3.29%
12	200	11.18	1020	180	205.87	2.94%
13	200	10.88	1010	665	196.76	1.62%
14	300	14.28	−80	250	300.24	0.11%
15	300	13.73	−70	680	283.30	5.57%
16	300	14.42	640	250	304.25	1.42%
17	300	13.91	630	660	288.77	3.74%
18	300	14.63	1090	270	310.63	3.54%
19	300	14.06	1020	650	293.32	2.23%

**Table 4:** Compensation coefficient for three-dimensional force fitting.

Parameter	a1	a2	a3	b1	b2
Value	−0.86	−42.59	3.63	−16.28	−74.27
**Parameter**	**b3**	**k0**	**k1**	**k2**	
Value	−40.61	291.45	−0.09	0.54	

**Table 5:** Results of three-dimensional force-fitting measurement.

No.	Mass (kg)	Pre Equilibrium Measured Data (kg)	Measurement Error before Balancing	Data Measured after Equalization (kg)	Measurement Error after Equalization
1	0	0.00	0%	0	0.00%
2	100	99.90	0.10%	97.49	2.51%
3	100	94.13	5.87%	98.09	1.91%
4	100	102.94	2.94%	99.13	0.87%
5	100	97.47	2.53%	99.41	0.59%
6	100	102.94	2.94%	97.51	2.49%
7	100	100.51	0.51%	100.71	0.71%
8	200	198.89	0.56%	199.06	0.47%
9	200	190.08	4.96%	198.10	0.95%
10	200	204.66	2.33%	198.04	0.98%
11	200	193.42	3.29%	196.95	1.53%
12	200	205.87	2.94%	195.90	2.05%
13	200	196.76	1.62%	197.41	1.30%
14	300	300.24	0.11%	299.67	0.11%
15	300	283.30	5.57%	294.60	1.80%
16	300	304.25	1.42%	296.92	1.03%
17	300	288.77	3.74%	293.80	2.07%
18	300	310.63	3.54%	299.14	0.29%
19	300	293.32	2.23%	294.25	1.92%

**Table 6:** Particle swarm optimization compensation coefficient.

Parameter	a1	a2	a3	b1	b2
Value	5.47	−38.39	13.31	−2.32	−63.92
**Parameter**	**b3**	**K0**	**K1**	**K2**	
Value	−32.25	300.69	−0.08	0.17	

**Table 7:** Particle swarm optimization standard weight measurement data.

No.	Mass (kg)	Pre Equilibrium Measured Data (kg)	Measurement Error before Balancing	Data Measured after Equalization (kg)	Measurement Error after Equalization
1	0	0	0.00%	0	0.00%
2	100	97.49	2.51%	99.65	0.35%
3	100	98.09	1.91%	99.44	0.56%
4	100	99.13	0.87%	101.23	1.23%
5	100	99.41	0.59%	101.23	1.23%
6	100	97.51	2.49%	98.89	1.11%
7	100	100.71	0.71%	101.23	1.23%
8	200	199.06	0.47%	200.69	0.34%
9	200	198.10	0.95%	199.39	0.31%
10	200	198.04	0.98%	200.80	0.40%
11	200	196.95	1.53%	198.98	0.51%
12	200	195.90	2.05%	197.67	1.17%
13	200	197.41	1.30%	198.68	0.66%
14	300	299.67	0.11%	302.39	0.80%
15	300	294.60	1.80%	298.48	0.51%
16	300	296.92	1.03%	299.58	0.14%
17	300	293.80	2.07%	296.91	1.03%
18	300	299.14	0.29%	299.35	0.22%
19	300	294.25	1.92%	296.30	1.23%

**Table 8:** Random point mass and position parameter.

No.	Mass (kg)	Eccentric Load Distance in *X* Direction (mm)	Eccentric Load Distance in *Y* Direction (mm)
1	150	1050	590
2	150	660	540
3	150	120	480
4	250	220	530
5	250	800	500
6	250	1150	560

**Table 9:** Measurements for three calculation methods (unit: kg).

No.	Mass	Eccentric Load Compensation	Three-Dimensional Force Biased Load Compensation	PSO Imbalance Compensation
1	150	148.79	151.27	150.68
2	150	147.87	151.65	150.44
3	150	146.25	151.60	149.27
4	250	242.91	252.51	250.70
5	250	247.77	251.39	249.50
6	250	247.16	250.82	249.21

**Table 10:** Measurement error for the three calculation methods.

No.	Eccentric Load Compensation	Three-Dimensional Force Biased Load Compensation	PSO Imbalance Compensation
1	0.81%	0.85%	0.45%
2	1.42%	1.10%	0.29%
3	2.63%	1.07%	0.49%
4	2.83%	1.00%	0.28%
5	0.89%	0.56%	0.20%
6	1.13%	0.33%	0.32%

## Data Availability

Data are unavailable due to privacy restrictions.
